# Frontoparietal Response to Working Memory Load Mediates the Association between Sleep Duration and Cognitive Function in Children

**DOI:** 10.3390/brainsci14070706

**Published:** 2024-07-14

**Authors:** Jie Yan, Haolei Bai, Yuqing Sun, Xueqi Sun, Zhian Hu, Bing Liu, Chao He, Xiaolong Zhang

**Affiliations:** 1 Department of Physiology, Institute of Brain and Intelligence, Third Military Medical University, Chongqing 400038, China; 2 State Key Laboratory of Cognitive Neuroscience and Learning, Beijing Normal University, Beijing 100875, China; 3 Chinese Institute for Brain Research, Beijing 102206, China

**Keywords:** sleep duration, frontoparietal activation, working memory, cognitive function, mediation analysis, children, sex difference

## Abstract

Lack of sleep has been found to be associated with cognitive impairment in children, yet the neural mechanism underlying this relationship remains poorly understood. To address this issue, this study utilized the data from the Adolescent Brain Cognitive Development (ABCD) study (*n* = 4930, aged 9–10), involving their sleep assessments, cognitive measures, and functional magnetic resonance imaging (fMRI) during an emotional n-back task. Using partial correlations analysis, we found that the out-of-scanner cognitive performance was positively correlated with sleep duration. Additionally, the activation of regions of interest (ROIs) in frontal and parietal cortices for the 2-back versus 0-back contrast was positively correlated with both sleep duration and cognitive performance. Mediation analysis revealed that this activation significantly mediated the relationship between sleep duration and cognitive function at both individual ROI level and network level. After performing analyses separately for different sexes, it was revealed that the mediation effect of the task-related activation was present in girls (*n* = 2546). These findings suggest that short sleep duration may lead to deficit in cognitive function of children, particularly in girls, through the modulation of frontoparietal activation during working memory load.

## 1. Introduction

Sleep is a critical component of the developmental framework, essential for fostering cognitive advancement and neural maturation [1,2,3]. Adherence to sufficient sleep duration has been shown to be positively associated with enhanced academic achievement in children [4]. Conversely, insufficient sleep in children has been linked to deficits in higher-order and complex cognitive functions [5], as well as a reduction in overall intelligence quotient score [6,7]. Given that insufficient sleep is prevalent among children and adolescents worldwide, a comprehensive understanding of the neural mechanisms underlying the effects of sleep duration on cognition is meaningful. In fact, several neuroimaging studies have shed light on the brain structures and functional connections that underpin the sleep duration–cognition relationship, such as the volume of the prefrontal cortex, temporal cortex, and medial orbitofrontal cortex [8], as well as the resting-state cortico-basal ganglia functional connections [9]. However, these studies primarily focused on the brain’s intrinsic functional organization. Task-based functional magnetic resonance imaging (fMRI), on the other hand, allows us to observe the brain’s dynamic responses that is specific to certain cognitive challenges [10,11], providing a more comprehensive understanding of how sleep affects cognitive function.

The activity of the frontal and parietal cortices has been found to be crucially involved in various cognitive tasks [12,13,14]. Specifically, frontoparietal activation supports working memory, which is the capacity to hold and manipulate information over a short period [15]. Working memory serves as a fundamental component of cognitive function, contributing to a wide range of mental activities necessary for complex thought and behavior [16,17]. In children, working memory is particularly important, as it is a domain of developmentally sensitive plasticity [18,19] and underpins many aspects of intellectual activities [20,21]. Furthermore, in a large sample study of children, frontoparietal activation during a working memory task was related to performance in a series of cognitive tasks, such as the NIH Toolbox Picture Vocabulary and Oral Reading Recognition tasks and the WISCV matrix reasoning test [22]. Recently, the frontoparietal response to a working memory task was also found to be a good predictor of cognitive ability in children [23]. Thus, frontoparietal activation during working memory challenge seems to play a critical role in the general cognitive performance of children.

Given the established importance of frontoparietal activation, it is noteworthy that this neural response can be influenced by external factors such as sleep loss [24,25,26]. After 24 and 35 h of sleep deprivation in healthy young adults, there was less recruitment of the superior parietal regions during a set of working memory tasks, paralleled by a decline in in-scanner performance [27]. A similar decrease in parietal activation during an n-back working memory task was also observed in adolescents under a week of chronic sleep restriction [28]. The observed decline in neural activity upon sleep loss underscores the pivotal role of sleep in maintaining optimal conditions for frontoparietal activation. Collectively, these data imply a triadic link between sleep, cognitive function, and the recruitment of frontal and parietal cortices during working memory challenge. However, to our knowledge, this model has not yet been tested.

In the current study, we hypothesized that changes in the frontoparietal response during working memory load would contribute to the effect of sleep duration on cognitive function. Specifically, it was predicted that the decreased sleep duration may reduce frontoparietal activation, leading to poorer cognitive performance. Using the baseline data from the Adolescent Brain and Cognitive Development (ABCD) Study [29], we first examined the pairwise associations between sleep assessments, frontoparietal activation during an n-back working memory task, and the cognitive performance. Then, we utilized mediation analysis to explore the underlying mediators between sleep duration and cognitive performance.

## 2. Materials and Methods

### 2.1. Participants

The baseline data of ABCD Study Data Release 4.0 (https://abcdstudy.org/index.html, accessed on 15 January 2024) was used for data analysis. The ABCD Study is a large-scale longitudinal study of adolescent neurocognitive development, recruiting 11,875 children aged 9–10 years from 21 sites across the United States [30]. The study involves annual multimodal assessments, including physical health and neurocognitive measures, complemented by structural and functional MRI scans conducted every two years. We used the baseline data, as it provides a substantial number of valid cases for the composite measure that reflects overall cognitive functioning. We excluded children who (a) showed signs of clinical referral in their MR findings, had a history of epilepsy, multiple sclerosis, and sickle cell anemia, or were diagnosed with cerebral palsy, tumor, stroke, brain aneurysm, brain hemorrhage, subdural hematoma, schizophrenia, autism spectrum disorder, intellectual disability, or other psychological or psychiatric disorders, to avoid confounding effects of underlying neurological conditions [31]; (b) scored below 60% accuracy in the task, to ensure reliable task performance data; (c) had fewer than two scan runs that passed image quality control and had mean framewise displacement < 0.9 mm, to ensure the quality and reliability of MRI data; (d) had extreme values (>3 SDs from the group mean) for task accuracy or beta weights of brain area, to eliminate outliers that could skew the analysis; (e) missed information about age, sex, race, puberty, parent education, scanner type, study site, sleep assessments, or cognition score, to ensure completeness of the data. For families with multiple children included, we randomly selected one child from each family, to ensure independent participants in the analyses. The final sample size was 4930 participants (see Table 1 for demographic characteristics). When conducting the study separately for boys and girls, if several participants of the same sex from a single household qualified for the study, only one was randomly chosen for inclusion (see Table S1 for sample sizes and demographic characteristics).

### 2.2. Sleep Assessments

The Parent-reported Sleep Disturbance Scale for Children was used to assess the sleep disorders in children [33]. The scale covers six different sleep disorders: disorders of initiating and maintaining sleep, sleep breathing disorders, disorder of arousal, sleep–wake transition disorders, disorders of excessive somnolence, and sleep hyperhidrosis. Sleep duration was measured by the individual item from the above scale, “How many hours of sleep does your child get on most nights in the past six months?”, with five possible response options. We recoded the responses so that a high score indicated long sleep duration (1 = less than 5 h, 2 = 5–7 h, 3 = 7–8 h, 4 = 8–9 h, 5 = 9–11 h).

### 2.3. Cognitive Function

Cognitive performance was assessed by the summary score of the NIH Cognition Battery Toolbox [34,35]. The NIH Cognition Battery Toolbox contains seven different tasks that cover language abilities, processing speed, working memory, episodic memory, attention, cognitive flexibility, and executive function.

### 2.4. Emotional n-Back Task

The emotional n-back (EN-back) task is a variant of a traditional n-back task performed in an fMRI scanner [36]. The task was designed to investigate working memory and emotional processes using four types of images (happy face, fearful face, neutral face, and place) with two memory load conditions (0 and 2 back) [29,37]. During the task, participants completed two approximately 5 min runs. In each run, there were four “0-back” blocks (low memory load) and four “2-back” blocks (high memory load). Each block consisted of 10 trials. Of the 10 trials, two were target stimulus, two or three were nontarget lures, and the rest of the trials were nonlures that presented only once. Across the entire experiment, participants encountered a total of 160 trials, utilizing 96 distinct images categorized into four distinct types (24 images per type). A total of 75% of the images were human faces displaying happy, fearful, or neutral expressions, with the type of facial expression remaining consistent within each block. For each trial, children were asked to indicate whether the current stimulus was identical to a target stimulus. In the 0-back condition, the target image was shown at the beginning of the block. In the 2-back condition, the target image was presented two trials back. Performance was assessed by the overall rate of correct responses during the 2-back condition.

### 2.5. Neuroimaging Data

**Figure 1:**
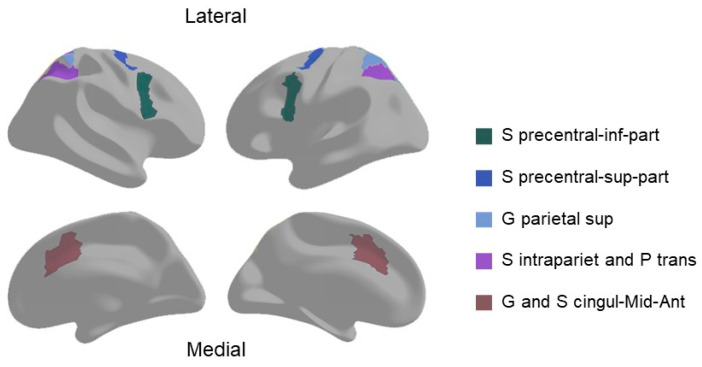
The locations of regions of interest (ROIs) for the 2-back versus 0-back contrast. Abbreviations: Inferior part of the precentral sulcus (S precentral-inf-part); superior part of the precentral sulcus (S precentral-sup-part); middle-anterior part of the cingulate gyrus and sulcus (G and S cingul-Mid-Ant); intraparietal sulcus and transverse parietal sulci (S intrapariet and P trans); superior parietal lobule (G parietal sup).

The full details of the ABCD scanning protocol and image processing pipelines have been previously described in detail [29,38]. In brief, task-based functional images were collected on 3T scanners (Siemens Prisma, General Electric 750 and Philips) with a multiband gradient echo-planar imaging sequence. Task-fMRI-specific preprocessing included the removal of initial volumes (8 TRs for Siemens and Philips, 5 TRs for GE DV25, and 16 TRs for GE DV26), and normalization and demeaning of voxel data. Task-related activation strength was estimated using a general linear model (GLM) in AFNI’s 3dDeconvolve with nuisance regressors for baseline, quadratic trend, and motion. The hemodynamic response function was modeled using gamma functions with temporal derivatives through AFNI’s SPMG model. GLM coefficients and *t*-statistics were subsequently sampled onto the cortical surface, projecting 1 mm into the cortical gray matter along surface normal vector. For the EN-back task, predictors were established for each type of stimulus across different n-back conditions and fixation. Linear contrasts were computed for various comparisons, such as 2-back versus 0-back across stimulus types (to measure the responses to working memory load), and emotional faces versus neutral faces across memory loads (to measure the responses to emotional stimuli). Regions of interest (ROIs) involving cortical areas were delineated according to the Destrieux atlas. Based on previous research, we selected the ROIs most likely recruited by working memory challenge in n-back task [29,39,40]: inferior part of the precentral sulcus (S precentral-inf-part), superior part of the precentral sulcus (S precentral-sup-part), middle-anterior part of the cingulate gyrus and sulcus (G and S cingul-Mid-Ant), intraparietal sulcus and transverse parietal sulci (S intrapariet and P trans), and superior parietal lobule (G parietal sup) (Figure 1). The averaged beta weights across two runs for each of these ROIs were used in follow-up analyses.

### 2.6. Statistical Analyses

Association analysis: Partial correlation analyses were conducted in Statistical Package for the Social Sciences 26 (SPSS 26, https://www.ibm.com/spss, accessed on 20 January 2024) to investigate the pairwise relationships between sleep assessments, cognitive performance, and the beta weights of ROIs. Age, sex, race, puberty, highest level of parental education, scanner type, study site, and average head motion were included as covariates [8,29]. False discovery rate (FDR) was performed to correct for multiple comparisons [41]. The correlation with *p*-value below the 0.05 threshold was considered significant. To visualize the brain activation patterns, we used BrainNet Viewer 1.41, a widely utilized tool for generating topographical maps of neuroimaging data [42].

Factor analysis: To assess frontoparietal activity at the network level, we conducted a principal components analysis (PCA) on the activation of the above 10 ROIs for the 2-back versus 0-back contrast utilizing SPSS. The Kaiser–Meyer–Olkin (KMO) measure of sampling adequacy was higher than the suggested minimum of 0.6, and the *p*-value from Bartlett’s test of sphericity was less than 0.001, indicating that the data were suitable for PCA. Then, components with an eigenvalue greater than 1 were retained for further analysis, and a loading value of 0.4 or higher was used as the criterion for variables to determine their loading onto the corresponding components. Finally, a varimax rotation was applied to simplify the interpretation of the factors.

Mediation analysis: We performed mediation analyses using a standard three-variable path model from the mediation toolbox developed by Tor Wager’s group (https://github.com/canlab/MediationToolbox, accessed on 20 January 2024). In the model, the independent variable was sleep duration, and the dependent variable was 2-back accuracy or cognition score. The proposed mediator was the beta weights of each ROI for the 2-back versus 0-back contrast or the network-level response derived from PCA. The eight covariates used in the association analysis were also controlled in the mediation model. The significance of the mediation was assessed via the bias-corrected bootstrap method with 10,000 random samplings.

## 3. Results

### 3.1. Association between Sleep Assessments and Cognitive Function

**Figure 2:**
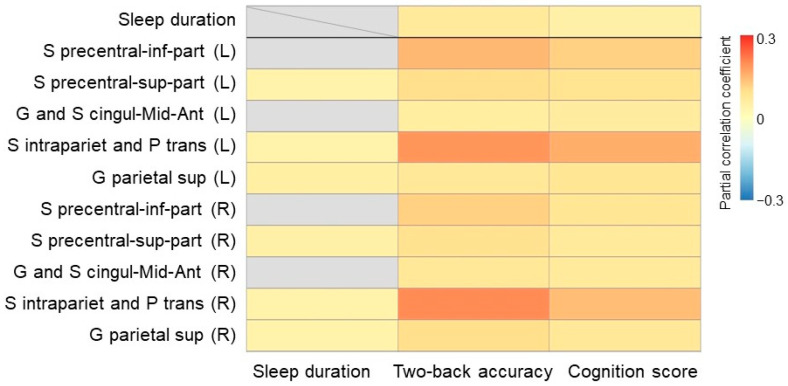
The pairwise associations between sleep duration, 2-back accuracy, cognition score, and the activation of ROIs for the 2-back versus 0-back contrast. The gray grid indicates a nonsignificant correlation (*p* > 0.05 FDR corrected). Abbreviations: left (L); right (R).

We first validated the association between sleep duration and cognitive function. The sleep duration was positively correlated with 2-back accuracy (*p* < 0.001, r = 0.068 (Figure 2)), as well as the cognition score (*p* = 0.001, r = 0.046 (Figure 2)). We next examined the specificity of our findings by correlating behavioral measures and the scores of six sleep disorders (disorders of initiating and maintaining sleep, sleep breathing disorders, disorder of arousal, sleep–wake transition disorders, disorders of excessive somnolence, and sleep hyperhidrosis). None of these sleep disorders showed a relationship with 2-back accuracy or cognition score (all *p* > 0.05, FDR corrected, Table S2).

### 3.2. Association between Sleep Duration and EN-Back Neural Activity

We then tested the association of sleep duration with brain activation during working memory load. Among the 10 ROIs examined, 6 ROIs exhibited higher mean beta weights for the 2-back versus 0-back contrast in children with longer sleep duration: bilateral S precentral-sup-part (left, *p* = 0.015, r = 0.037; right, *p* = 0.005, r = 0.047, FDR corrected (Figure 2 and Figure S1)), bilateral S intrapariet and P trans (left, *p* = 0.012, r = 0.039; right, *p* = 0.011, r = 0.040), and bilateral G parietal sup (left, *p* = 0.005, r = 0.049; right, *p* = 0.011, r = 0.041). In order to explore whether the impact of sleep duration on the ROIs activation was specific to working memory stimuli, we also assessed the association of sleep duration with the ROIs activation for the contrasts of face versus place and emotional versus neutral face. There was no significant correlation between sleep duration and the ROIs activation for either of the two contrasts (all *p* > 0.05, FDR corrected, Table S3).

### 3.3. Association between EN-Back Neural Activity and Cognitive Function

The activation of the 10 ROIs for the 2-back versus 0-back contrast was all positively correlated with 2-back accuracy (S precentral-inf-part, left, *p* < 0.001, r = 0.150; right, *p* < 0.001, r = 0.117; S precentral-sup-part, left, *p* < 0.001, r = 0.100; right, *p* < 0.001, r = 0.094; G and S cingul-Mid-Ant, left, *p* < 0.001, r = 0.057; right, *p* < 0.001, r = 0.075; S intrapariet and P trans, left, *p* < 0.001, r = 0.189; right, *p* < 0.001, r = 0.202; G parietal sup, left, *p* < 0.001, r = 0.077; right, *p* < 0.001, r = 0.101, FDR corrected (Figure 2 and Figure S2)). Similarly, a significant positive association was also found between the 10 ROIs activation for the 2-back versus 0-back contrast and the cognition score (S precentral-inf-part, left, *p* < 0.001, r = 0.116; right, *p* < 0.001, r = 0.078; S precentral-sup-part, left, *p* < 0.001, r = 0.089; right, *p* < 0.001, r = 0.069; G and S cingul-Mid-Ant, left, *p* < 0.001, r = 0.063; right, *p* < 0.001, r = 0.071; S intrapariet and P trans, left, *p* < 0.001, r = 0.157; right, *p* < 0.001, r = 0.140; G parietal sup, left, *p* < 0.001, r = 0.079; right, *p* < 0.001, r = 0.078, FDR corrected (Figure 2 and Figure S3)). In contrast, neither of the ROIs activation for the contrast of face versus place or emotional versus neutral face was significantly associated with 2-back accuracy or cognition score (all *p* > 0.05, FDR corrected (Table S4)).

### 3.4. Mediation Analysis

**Figure 3:**
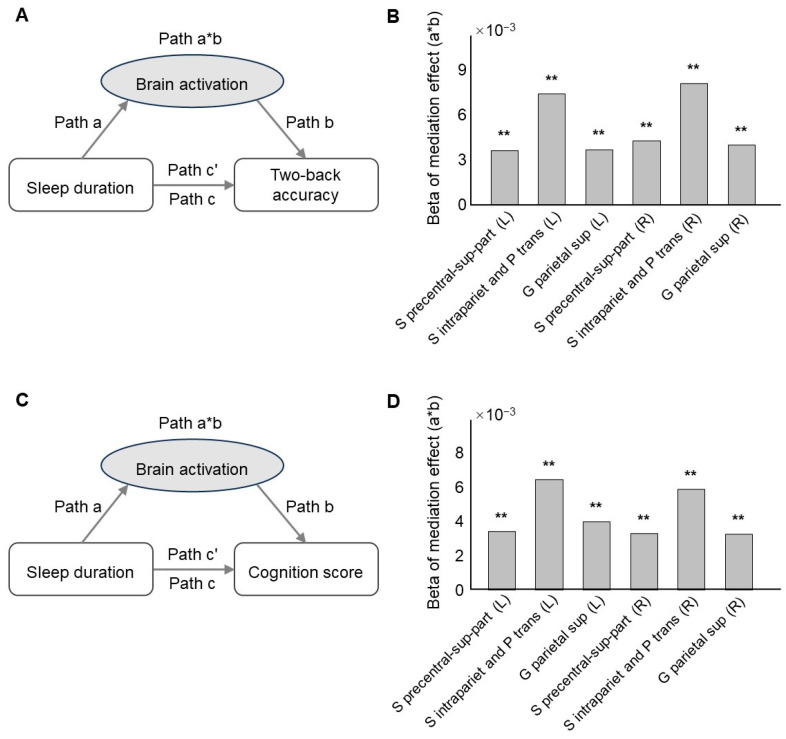
(**A**) The mediation model testing whether the activation of the ROIs in the 2-back versus 0-back contrast mediated the effect of sleep duration on 2-back accuracy. (**B**) Mediation effects of the ROIs activation in the model presented in panel (**A**). (**C**) The mediation model testing whether the activation of ROIs in the 2-back versus 0-back contrast mediated the effect of sleep duration on cognition score. (**D**) Mediation effects of the ROIs activation in the model presented in panel (**C**). Only the ROIs for which the activation during the 2-back versus 0-back contrast showed significant correlations with both sleep duration and cognition score are presented. ** *p*_FDR_ < 0.01.

To explore whether the activation of ROIs acted as a mediator between sleep duration and behavioral measures, the six ROIs for which the activation showed significant correlations with both sleep duration and behavioral measures were examined in the mediation model. All of them were found to significantly mediate the effect of sleep duration on 2-back accuracy (S precentral-sup-part, left, *p* = 0.007, β = 3.65 × 10^−3^; right, *p* = 0.001, β = 4.27 × 10^−3^; S intrapariet and P trans, left, *p* = 0.006, β = 7.39 × 10^−3^; right, *p* = 0.006, β = 8.12 × 10^−3^; G parietal sup, left, *p* = 0.001, β = 3.69 × 10^−3^; right, *p* = 0.006, β = 4.02 × 10^−3^, FDR corrected (Figure 3A,B, Table S5)), as well as the effect of sleep duration on cognition score (S precentral-sup-part, left, *p* = 0.007, β = 3.42 × 10^−3^; right, *p* = 0.001, β = 3.30 × 10^−3^; S intrapariet and P trans, left, *p* = 0.006, β = 6.44 × 10^−3^; right, *p* = 0.004, β = 5.89 × 10^−3^; G parietal sup, left, *p* = 0.001, β = 3.98 × 10^−3^; right, *p* = 0.004, β = 3.28 × 10^−3^, FDR corrected (Figure 3C,D, Table S6)).

### 3.5. Network-Level Response

**Figure 4:**
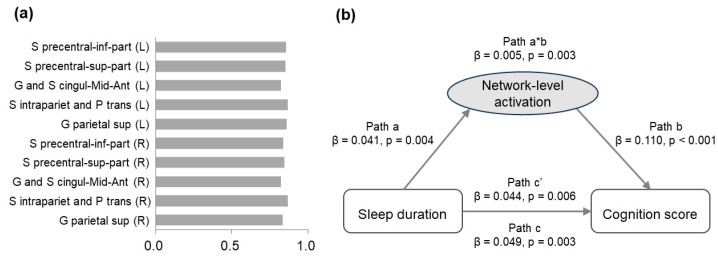
(**a**) Loadings for the activation of each ROI in the 2-back versus 0-back contrast. (**b**) The mediation model showing the association between sleep duration and cognition score as mediated by the frontoparietal activation at the network level.

To examine the mediation effect of task-related frontoparietal activation at the network level, factor analysis was conducted on the activation of the 10 ROIs for the 2-back versus 0-back contrast. From the PCA, we extracted one component (see Figure 4a for component loadings), which accounted for 71.64% of the total variance. This component significantly mediated the association between sleep duration and cognition score (*p* = 0.003, β = 4.53 × 10^−3^ (Figure 4b)).

### 3.6. Sex Effects

**Figure 5:**
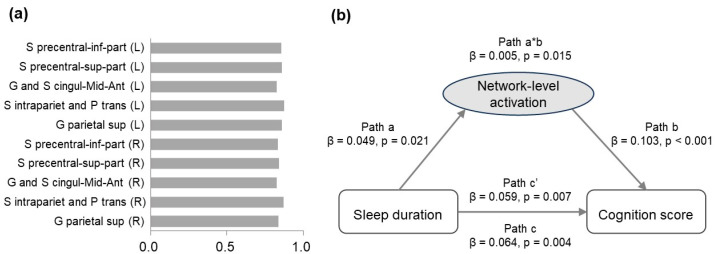
(**a**) Loadings for the activation of each ROI in the 2-back versus 0-back contrast in girls. (**b**) The mediation model in girls showing the association between sleep duration and cognition score as mediated by the frontoparietal activation at the network level.

We performed a stratified analysis to test whether the mediation effect of task-related frontoparietal activation existed in both boys and girls. A significant relationship between sleep duration and cognition score was found in girls (*n* = 2546, *p* = 0.002, r = 0.061), but not in boys (*n* = 2527, *p* = 0.117). Using PCA on the activation of the 10 ROIs for the 2-back versus 0-back contrast in girls, one component was extracted (see Figure 5a for component loadings), which accounted for 72.07% of the total variance. This component was positively correlated with both sleep duration (*p* = 0.020, r = 0.046) and cognition score (*p* < 0.001, r = 0.106) of girls, and significantly mediated the effect of sleep duration on cognition score (*p* = 0.015, β = 5.06 × 10^−3^ (Figure 5b)).

## 4. Discussion

The present study investigates the potential neural mechanism linking sleep duration to cognitive function in children. Our findings indicate that the parent-reported sleep duration positively correlated with in-scanner 2-back accuracy, out-of-scanner cognitive performance, and frontoparietal activation in the working memory task. Furthermore, cognitive performance correlated positively with task-related frontoparietal activation. Mediation analysis revealed that the task-related frontoparietal activation mediated the influence of sleep duration on cognitive performance at both individual ROIs level and network level. Notably, a stratified analysis showed that the mediation effect of task-related frontoparietal activation existed in girls. These results together suggest that the frontoparietal response to working memory load may act as a mediator between sleep duration and cognitive function, especially for girls.

The link between sleep duration and frontoparietal activation in the 2-back versus 0-back contrast is consistent with previous studies, which demonstrated a decrease in the recruitment of parietal regions during working memory tasks after acute sleep deprivation or chronic sleep restriction [27,28]. However, unlike previous research, which involved experimental manipulation of sleep, we investigated the effects of sleep duration under naturalistic conditions. Thus, our findings underscore the importance of adequate sleep for optimizing neural activation in daily life. One possible explanation for the effect of sleep duration on task-related frontoparietal activation is that sleep state is an active process that restores synaptic and cellular homeostasis [43,44]. Therefore, longer sleep duration may benefit the restoration of the capacity of the frontoparietal region to encode new information. On the other hand, we found no significant correlation between sleep duration and the frontoparietal activation for the contrasts of face versus place or emotional versus neutral face. However, it is widely known that sleep is associated with emotional outcomes in both experimental and observational studies [45]. The short sleep duration has been linked to decreased amygdala reactivity to negative facial expressions [46]. The negative results in our study may be due to the fact that our ROIs are not included in the core face-processing network [47], implying that these ROIs are not sensitive to emotional stimuli as amygdala.

In this study, we also observed a positive association between the frontoparietal response to working memory load and general cognitive performance. This finding was expected, given the supporting role of working memory in higher cognitive function [16,17], and is supported by recent research indicating that frontoparietal activation during n-back task can serve as a predictor of cognitive abilities in both children and young adults [23,48]. Moreover, our results suggest that there is no notable correlation between cognitive performance and the response of the ROIs to social stimuli (the contrast of face versus place) or to emotional stimuli (the contrast of emotional versus neutral face). These results imply that the frontoparietal activation in response to working memory challenge, rather than other task demands, contributes to general cognitive function.

Plenty of studies have identified the impact of sleep duration on cognitive function in children [5,6,7]. Previous studies mainly focused on brain structure and resting-state functional connection as explanatory mechanisms for this association. To be specific, short sleep duration may lead to lower cortical volume and less corticobasal ganglia connection in the resting state, resulting in poor cognitive performance [8,9]. Our study confirmed the association between sleep duration and cognitive performance. In addition, we found that the frontoparietal response to working memory load was a significant mediator for this association. These data provide a novel insight from the perspective of task-related brain recruitment, highlighting the dynamic shifts in neural engagement that occur during cognitive challenges, and, thus, may advance our understanding of how sleep impacts cognitive function. Moreover, as nearly half of the subjects in the study did not achieve the recommended amount of sleep [32], these results suggest that insufficient sleep may disrupt the normal functioning of brain regions critical for cognitive processing. Given these considerations, our study reinforces the message that ensuring adequate sleep is essential for supporting cognitive health in children. It also advocates for interventions that prioritize healthy sleep habits to promote optimal brain function in children. From a practical perspective, educational programs targeting both parents and children could raise awareness of the impact of sleep loss and encourage better sleep practices [49]. Leveraging mobile apps to monitor sleep patterns and provide personalized sleep improvement plans may offer helpful solutions to pediatric sleep issues [50].

Our findings revealed a sex difference in the relationship between sleep duration and cognitive function, with a significant correlation observed in girls but not in boys. This sex disparity aligns with previous research reporting that females are more vulnerable to cognitive deficits caused by sleep loss [51,52,53]. One possible explanation for this difference is the hormonal influence. Studies have shown that hormonal levels begin to fluctuate from preadolescence, and lead to the sex difference in the development of brain structure and brain function [54]. These hormones may also affect the sleep-related cognitive outcomes. Another possible explanation is the developmental disparities between boys and girls, including variations in brain maturation rates and cognitive development trajectories [55,56]. The differential maturation may contribute to the varying sensitivity to sleep loss observed in our study. Furthermore, in girls, we also found a mediation effect of the task-related frontoparietal activation on the relationship between sleep duration and cognitive performance. This highlights the potential role of neural activation in the impact of sleep on cognitive function again, and emphasizes the need for considering sex differences in future research on sleep and cognition in children.

The current study has several limitations. First, there may be a discrepancy between the parent-reported sleep duration and the actual sleep duration of children [57]. Parental reports, though convenient, may overestimate sleep duration and do not fully capture the child’s sleep experience. This limitation underscores the need for more objective sleep measurement techniques, such as actigraphy, which can provide a detailed and continuous record of sleep–wake patterns in the child’s natural environment. The incorporation of actigraphy in future study will not only enhance the precision of sleep measurement but also allow for a more nuanced understanding of sleep characteristics, including sleep architecture and the impact of social jetlag, which may have their own distinct effects on cognitive function [58]. Second, several neuroimaging studies have shown compensatory cerebral responses following sleep deprivation, which help to sustain cognitive performance [59,60]. Our study did not address compensatory activations that might occur when sleep duration is shorter. Further studies should consider examining these compensatory mechanisms to expand our knowledge of the cognitive impairment associated with insufficient sleep. Third, our exclusion criteria may introduce certain biases into the study. For instance, excluding children with neurological or psychiatric conditions means that our findings may not be generalizable to populations with these conditions. These factors should be considered when interpreting the results and generalizing findings to broader populations. Moreover, the neural substrates associated with cognitive function may vary across different developmental stages [61]. As our findings are based on a specific age group, caution should be exercised when extrapolating these results to other populations. Future research should include diverse age groups and populations to strengthen the external validity of the conclusions. Additionally, future studies should incorporate longitudinal designs to track changes in sleep patterns and cognitive function over time, offering an elaborate understanding of the long-term impacts of sleep duration on children’s cognitive development.

## 5. Conclusions

This study proposes that the frontoparietal activation during working memory challenge could be a potential neural mechanism mediating the impact of reduced sleep duration on cognitive performance, especially in girls. These findings contribute to a more comprehensive understanding of the role of sleep in cognitive function, and emphasize the importance of sufficient sleep for children to develop their frontoparietal function.

## Figures and Tables

**Table 1:** Demographic characteristics of the sample.

Variable	Mean (or N)	SD (or %)
Age (month)	119.53	7.47
Sex		
Male	2464	50.0
Female	2466	50.0
Race/Ethnicity		
White	2877	58.4
Black	475	9.6
Hispanic	945	19.2
Asian	117	2.4
Other	516	10.5
Pubertal development scale	1.73	0.86
Highest parent education	17.52	2.41
Scanner type		
GE	1275	25.9
Philips	533	10.8
Siemens	3122	63.3
Average motion (mm)	0.20	0.14
Two-back accuracy	0.80	0.09
Sleep duration		
1: Less than 5 h	5	0.1
2: 5–7 h	109	2.2
3: 7–8 h	484	9.8
4: 8–9 h	1759	35.7
5: 9–11 h *	2573	52.2
Cognition score	50.00	10.98

* The National Sleep Foundation’s sleep duration recommendation for children aged 9–10 years [32].

## Data Availability

Data are available at The National Institute of Mental Health Data Archive (NDA) accessible at https://abcdstudy.org/index.html, accessed on 15 January 2024.

## Supplementary Materials

The following supporting information can be downloaded at: https://www.mdpi.com/article/10.3390/brainsci14070706/s1, Figure S1: Association between sleep duration and the regions of interest (ROIs) activity for the 2-back versus 0-back contrast; Figure S2: Association between 2-back accuracy and the ROIs activity for the 2-back versus 0-back contrast; Figure S3: Association between cognition score and the ROIs activity for the 2-back versus 0-back contrast; Table S1: Demographic characteristics of the male and female samples; Table S2: Association between sleep assessments and behavioral measures; Table S3: Association between sleep duration and the ROIs activity for the contrasts of face versus place and emotional versus neutral face; Table S4: Association of 2-back accuracy and cognition score with the ROIs activity for the contrasts of face versus place and emotional versus neutral face; Table S5: The coefficients in the mediation model testing whether the ROIs activity for the 2-back versus 0-back contrast mediated the effect of sleep duration on 2-back accuracy; Table S6: The coefficients in the mediation model testing whether the ROIs activity for the 2-back versus 0-back contrast mediated the effect of sleep duration on cognition score.

## References

[1] Dutil C., Walsh J.J., Featherstone R.B., Gunnell K.E., Tremblay M.S., Gruber R., Weiss S.K., Cote K.A., Sampson M., Chaput J.P. (2018). Influence of sleep on developing brain functions and structures in children and adolescents: A systematic review. Sleep Med. Rev..

[2] de Bruin E.J., van Run C., Staaks J., Meijer A.M. (2017). Effects of sleep manipulation on cognitive functioning of adolescents: A systematic review. Sleep Med. Rev..

[3] Tarokh L., Saletin J.M., Carskadon M.A. (2016). Sleep in adolescence: Physiology, cognition and mental health. Neurosci. Biobehav. Rev..

[4] Bao R., Qin H., Memon A.R., Chen S., Lopez-Gil J.F., Liu S., Zou L., Cai Y. (2024). Is adherence to the 24-h movement guidelines associated with greater academic-related outcomes in children and adolescents? A systematic review and meta-analysis. Eur. J. Pediatr..

[5] Astill R.G., Van der Heijden K.B., Van Ijzendoorn M.H., Van Someren E.J. (2012). Sleep, cognition, and behavioral problems in school-age children: A century of research meta-analyzed. Psychol. Bull..

[6] Short M.A., Blunden S., Rigney G., Matricciani L., Coussens S., Reynolds C.M., Galland B. (2018). Cognition and objectively measured sleep duration in children: A systematic review and meta-analysis. Sleep Health.

[7] Gruber R., Laviolette R., Deluca P., Monson E., Cornish K., Carrier J. (2010). Short sleep duration is associated with poor performance on IQ measures in healthy school-age children. Sleep Med..

[8] Cheng W., Rolls E., Gong W., Du J., Zhang J., Zhang X.Y., Li F., Feng J. (2021). Sleep duration, brain structure, and psychiatric and cognitive problems in children. Mol. Psychiatr..

[9] Yang F.N., Xie W., Wang Z. (2022). Effects of sleep duration on neurocognitive development in early adolescents in the USA: A propensity score matched, longitudinal, observational study. Lancet Child. Adolesc. Health.

[10] Finn E.S., Poldrack R.A., Shine J.M. (2023). Functional neuroimaging as a catalyst for integrated neuroscience. Nature.

[11] Zhao W., Makowski C., Hagler D.J., Garavan H.P., Thompson W.K., Greene D.J., Jernigan T.L., Dale A.M. (2023). Task fMRI paradigms may capture more behaviorally relevant information than resting-state functional connectivity. Neuroimage.

[12] Niendam T.A., Laird A.R., Ray K.L., Dean Y.M., Glahn D.C., Carter C.S. (2012). Meta-analytic evidence for a superordinate cognitive control network subserving diverse executive functions. Cogn. Affect. Behav. Neurosci..

[13] Jung R.E., Haier R.J. (2007). The Parieto-Frontal Integration Theory (P-FIT) of intelligence: Converging neuroimaging evidence. Behav. Brain Sci..

[14] Ren X., Libertus M.E. (2023). Identifying the Neural Bases of Math Competence Based on Structural and Functional Properties of the Human Brain. J. Cogn. Neurosci..

[15] Luckmann H.C., Jacobs H.I., Sack A.T. (2014). The cross-functional role of frontoparietal regions in cognition: Internal attention as the overarching mechanism. Prog. Neurobiol..

[16] Chai W.J., Abd H.A., Abdullah J.M. (2018). Working Memory From the Psychological and Neurosciences Perspectives: A Review. Front. Psychol..

[17] Cowan N. (2014). Working Memory Underpins Cognitive Development, Learning, and Education. Educ. Psychol. Rev..

[18] Brehmer Y., Li S.C., Muller V., von Oertzen T., Lindenberger U. (2007). Memory plasticity across the life span: Uncovering children’s latent potential. Dev. Psychol..

[19] Fuhrmann D., Knoll L.J., Blakemore S.J. (2015). Adolescence as a Sensitive Period of Brain Development. Trends Cogn. Sci..

[20] Ikeda Y., Kita Y., Oi Y., Okuzumi H., Lanfranchi S., Pulina F., Mammarella I.C., Allen K., Giofre D. (2023). The Structure of Working Memory and Its Relationship with Intelligence in Japanese Children. J. Intell..

[21] Ger E., Roebers C.M. (2023). The Relationship between Executive Functions, Working Memory, and Intelligence in Kindergarten Children. J. Intell..

[22] Rosenberg M.D., Martinez S.A., Rapuano K.M., Conley M.I., Cohen A.O., Cornejo M.D., Hagler D.J., Meredith W.J., Anderson K.M., Wager T.D. (2020). Behavioral and Neural Signatures of Working Memory in Childhood. J. Neurosci..

[23] Pat N., Wang Y., Anney R., Riglin L., Thapar A., Stringaris A. (2022). Longitudinally stable, brain-based predictive models mediate the relationships between childhood cognition and socio-demographic, psychological and genetic factors. Hum. Brain Mapp..

[24] Chee M.W., Chuah L.Y. (2008). Functional neuroimaging insights into how sleep and sleep deprivation affect memory and cognition. Curr. Opin. Neurol..

[25] Ma N., Dinges D.F., Basner M., Rao H. (2015). How acute total sleep loss affects the attending brain: A meta-analysis of neuroimaging studies. Sleep.

[26] Yao L., Wang Y., Gao Y., Gao H., Guo X. (2023). The role of the fronto-parietal network in modulating sustained attention under sleep deprivation: An functional magnetic resonance imaging study. Front. Psychiatry.

[27] Chee M.W., Chuah L.Y., Venkatraman V., Chan W.Y., Philip P., Dinges D.F. (2006). Functional imaging of working memory following normal sleep and after 24 and 35 h of sleep deprivation: Correlations of fronto-parietal activation with performance. Neuroimage.

[28] Alsameen M., DiFrancesco M.W., Drummond S., Franzen P.L., Beebe D.W. (2021). Neuronal activation and performance changes in working memory induced by chronic sleep restriction in adolescents. J. Sleep Res..

[29] Casey B.J., Cannonier T., Conley M.I., Cohen A.O., Barch D.M., Heitzeg M.M., Soules M.E., Teslovich T., Dellarco D.V., Garavan H. (2018). The Adolescent Brain Cognitive Development (ABCD) study: Imaging acquisition across 21 sites. Dev. Cogn. Neurosci..

[30] Garavan H., Bartsch H., Conway K., Decastro A., Goldstein R.Z., Heeringa S., Jernigan T., Potter A., Thompson W., Zahs D. (2018). Recruiting the ABCD sample: Design considerations and procedures. Dev. Cogn. Neurosci..

[31] Kwon Y.H., Yoo K., Nguyen H., Jeong Y., Chun M.M. (2021). Predicting multilingual effects on executive function and individual connectomes in children: An ABCD study. Proc. Natl. Acad. Sci. USA.

[32] Hirshkowitz M., Whiton K., Albert S.M., Alessi C., Bruni O., DonCarlos L., Hazen N., Herman J., Katz E.S., Kheirandish-Gozal L. (2015). National Sleep Foundation’s sleep time duration recommendations: Methodology and results summary. Sleep Health.

[33] Bruni O., Ottaviano S., Guidetti V., Romoli M., Innocenzi M., Cortesi F., Giannotti F. (1996). The Sleep Disturbance Scale for Children (SDSC) Construction and validation of an instrument to evaluate sleep disturbances in childhood and adolescence. J. Sleep Res..

[34] Luciana M., Bjork J.M., Nagel B.J., Barch D.M., Gonzalez R., Nixon S.J., Banich M.T. (2018). Adolescent neurocognitive development and impacts of substance use: Overview of the adolescent brain cognitive development (ABCD) baseline neurocognition battery. Dev. Cogn. Neurosci..

[35] Akshoomoff N., Beaumont J.L., Bauer P.J., Dikmen S.S., Gershon R.C., Mungas D., Slotkin J., Tulsky D., Weintraub S., Zelazo P.D. (2013). NIH Toolbox Cognition Battery (CB): Composite scores of crystallized, fluid, and overall cognition. Monogr. Soc. Res. Child Dev..

[36] Barch D.M., Burgess G.C., Harms M.P., Petersen S.E., Schlaggar B.L., Corbetta M., Glasser M.F., Curtiss S., Dixit S., Feldt C. (2013). Function in the human connectome: Task-fMRI and individual differences in behavior. Neuroimage.

[37] Chaarani B., Hahn S., Allgaier N., Adise S., Owens M.M., Juliano A.C., Yuan D.K., Loso H., Ivanciu A., Albaugh M.D. (2021). Baseline brain function in the preadolescents of the ABCD Study. Nat. Neurosci..

[38] Hagler D.J., Hatton S., Cornejo M.D., Makowski C., Fair D.A., Dick A.S., Sutherland M.T., Casey B.J., Barch D.M., Harms M.P. (2019). Image processing and analysis methods for the Adolescent Brain Cognitive Development Study. Neuroimage.

[39] Kennedy J.T., Harms M.P., Korucuoglu O., Astafiev S.V., Barch D.M., Thompson W.K., Bjork J.M., Anokhin A.P. (2022). Reliability and stability challenges in ABCD task fMRI data. Neuroimage.

[40] Yaple Z., Arsalidou M. (2018). N-back Working Memory Task: Meta-analysis of Normative fMRI Studies With Children. Child Dev..

[41] Yoav B., Yosef H. (1995). Controlling the False Discovery Rate: A Practical and Powerful Approach to Multiple Testing. J. R. Stat. Soc. Ser. B (Methodol.).

[42] Xia M., Wang J., He Y. (2013). BrainNet Viewer: A network visualization tool for human brain connectomics. PLoS ONE.

[43] Tononi G., Cirelli C. (2006). Sleep function and synaptic homeostasis. Sleep Med. Rev..

[44] Tononi G., Cirelli C. (2014). Sleep and the price of plasticity: From synaptic and cellular homeostasis to memory consolidation and integration. Neuron.

[45] Baglioni C., Spiegelhalder K., Lombardo C., Riemann D. (2010). Sleep and emotions: A focus on insomnia. Sleep Med. Rev..

[46] Schiel J.E., Tamm S., Holub F., Petri R., Dashti H.S., Domschke K., Feige B., Lane J.M., Riemann D., Rutter M.K. (2022). Associations Between Sleep Health and Amygdala Reactivity to Negative Facial Expressions in the UK Biobank Cohort. Biol. Psychiatry.

[47] Muller V.I., Hohner Y., Eickhoff S.B. (2018). Influence of task instructions and stimuli on the neural network of face processing: An ALE meta-analysis. Cortex.

[48] Sripada C., Angstadt M., Rutherford S., Taxali A., Shedden K. (2020). Toward a “treadmill test” for cognition: Improved prediction of general cognitive ability from the task activated brain. Hum. Brain Mapp..

[49] Bonuck K., Collins-Anderson A., Schechter C.B., Felt B.T., Chervin R.D. (2022). Effects of a Sleep Health Education Program for Children and Parents on Child Sleep Duration and Difficulties: A Stepped-Wedge Cluster Randomized Clinical Trial. JAMA Netw. Open.

[50] Al M.A., Wu J., Mubin O. (2022). A scoping review of mobile apps for sleep management: User needs and design considerations. Front. Psychiatry.

[51] Hajali V., Andersen M.L., Negah S.S., Sheibani V. (2019). Sex differences in sleep and sleep loss-induced cognitive deficits: The influence of gonadal hormones. Horm. Behav..

[52] Rangtell F.H., Karamchedu S., Andersson P., Liethof L., Olaya B.M., Lampola L., Schioth H.B., Cedernaes J., Benedict C. (2019). A single night of sleep loss impairs objective but not subjective working memory performance in a sex-dependent manner. J. Sleep Res..

[53] Saksvik I.B., Bjorvatn B., Hetland H., Sandal G.M., Pallesen S. (2011). Individual differences in tolerance to shift work--a systematic review. Sleep Med. Rev..

[54] Gur R.E., Gur R.C. (2016). Sex differences in brain and behavior in adolescence: Findings from the Philadelphia Neurodevelopmental Cohort. Neurosci. Biobehav. Rev..

[55] Wierenga L.M., Langen M., Oranje B., Durston S. (2014). Unique developmental trajectories of cortical thickness and surface area. Neuroimage.

[56] Cornblath E.J., Tang E., Baum G.L., Moore T.M., Adebimpe A., Roalf D.R., Gur R.C., Gur R.E., Pasqualetti F., Satterthwaite T.D. (2019). Sex differences in network controllability as a predictor of executive function in youth. Neuroimage.

[57] Turan O., Garner J., Chang L., Isaiah A. Accuracy of parent-reported sleep duration among adolescents assessed using accelerometry. Pediatr. Res..

[58] Taillard J., Sagaspe P., Philip P., Bioulac S. (2021). Sleep timing, chronotype and social jetlag: Impact on cognitive abilities and psychiatric disorders. Biochem. Pharmacol..

[59] Strangman G., Thompson J.H., Strauss M.M., Marshburn T.H., Sutton J.P. (2005). Functional brain imaging of a complex navigation task following one night of total sleep deprivation: A preliminary study. J. Sleep Res..

[60] Cui J., Tkachenko O., Gogel H., Kipman M., Preer L.A., Weber M., Divatia S.C., Demers L.A., Olson E.A., Buchholz J.L. (2015). Microstructure of frontoparietal connections predicts individual resistance to sleep deprivation. Neuroimage.

[61] Zhang Z., Peng P., Eickhoff S.B., Lin X., Zhang D., Wang Y. (2021). Neural substrates of the executive function construct, age-related changes, and task materials in adolescents and adults: ALE meta-analyses of 408 fMRI studies. Dev. Sci..

