# Supplementary material for: Frontoparietal Response to Working Memory Load Mediates the Association between Sleep Duration and Cognitive Function in Children
Source: Brain Sci. 2024 Jul 14;14(7):706. doi: 10.3390/brainsci14070706 (PMC11274878; doi:10.3390/brainsci14070706)
Supplement: Supplementary file 1 [file brainsci-14-00706-s001.zip › brainsci-3082457-supplementary.pdf]

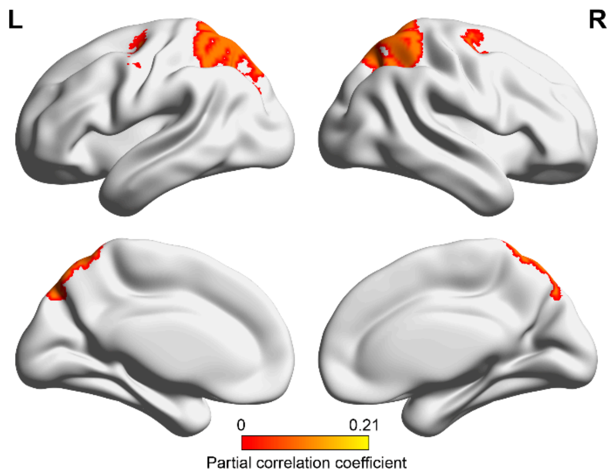

Figure S1. Association between sleep duration and the regions of interest (ROIs) activity for the 2-back versus 0-back contrast. Only the ROIs with False-discovery-rate-corrected P value (P_{FDR}) < 0.05 are presented.

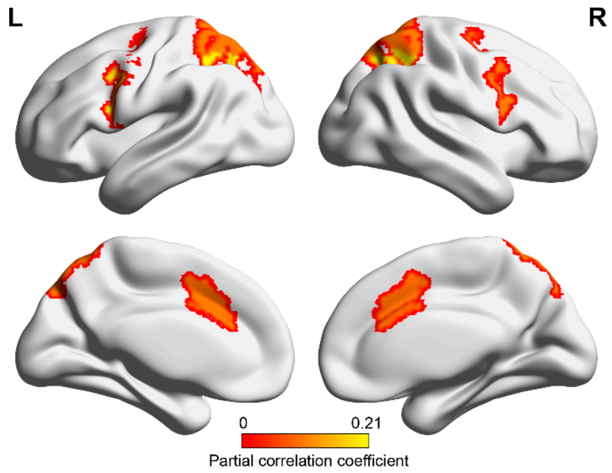

Figure S2. Association between 2-back accuracy and the ROIs activity for the 2-back versus 0-back contrast.

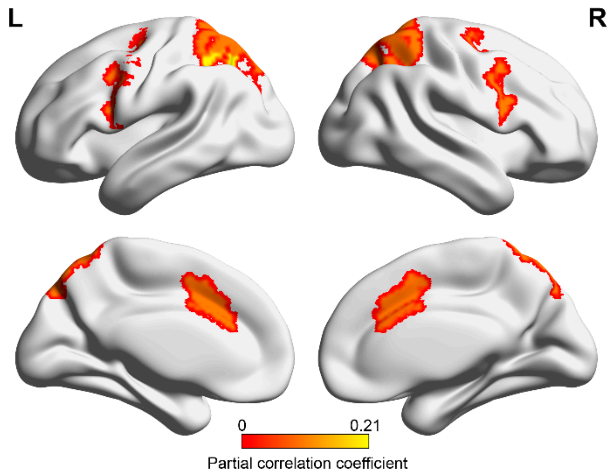

Figure S3. Association between cognition score and the ROIs activity for the 2-back versus 0-back contrast.

Table S1. Demographic characteristics of the male and female samples.

	Male (n = 2527)		Female (n = 2546)	
Variable	Mean (or N)	SD (or %)	Mean (or N)	SD (or %)
Age (month)	119.78	7.56	119.22	7.48
Race/Ethnicity				
White	1536	60.8	1433	56.3
Black	221	8.7	264	10.4
Hispanic	466	18.4	501	19.7
Asian	49	1.9	73	2.9
Other	255	10.1	275	10.8
Pubertal development scale	1.32	0.57	2.13	0.91
Highest parent education	17.56	2.38	17.50	2.43
Scanner type				
GE	659	26.1	649	25.5
Philips	274	10.8	269	10.6
Siemens	1594	63.1	1628	63.9
Average motion (mm)	0.22	0.14	0.19	0.13
Two-back accuracy	0.81	0.09	0.79	0.08
Sleep duration				
1: Less than 5 hours	1	0.0	4	0.2
2: 5-7 hours	57	2.3	54	2.1
3: 7-8 hours	250	9.9	244	9.6
4: 8-9 hours	878	34.7	925	36.3
5: 9-11 hours*	1341	53.1	1319	51.8
Cognition score	49.93	10.57	50.05	11.38

* The National Sleep Foundation's sleep duration recommendation for children aged 9-10 years.

Table S2. Association between sleep assessments and behavioral measures.

	Two-back accuracy		Cognition score	
	P_{FDR}	r	P_{FDR}	r
Disorders of initiating and maintaining sleep	0.2845	-0.0209	0.2355	-0.0271
Sleep breathing disorders	0.6635	-0.0062	0.2355	-0.0251
Disorder of arousal	0.2789	0.0239	0.9845	0.0003
Sleep-Wake transition disorders	0.6635	0.0063	0.2522	-0.0218
Disorders of excessive somnolence	0.3004	-0.0183	0.4519	-0.0126
Sleep Hyperhydrosis	0.0989	0.0342	0.3095	0.0180

Table S3. Association between sleep duration and the ROIs activity for the contrasts of face versus place and emotional versus neutral face.

Regions	Face versus place contrast	Emotional versus neutral face contrast
	P_{FDR}	P_{FDR}
S precentral-inf-par (L)	0.7758	0.8862
S precentral-sup-part (L)	0.7758	0.8862
G and S cingul-Mid-Ant (L)	0.7758	0.8862
S intrapariet and P trans (L)	0.7758	0.8862
G parietal sup (L)	0.7758	0.9372
S precentral-inf-par (R)	0.7758	0.8862
S precentral-sup-part (R)	0.7758	0.8862
G and S cingul-Mid-Ant (R)	0.7758	0.8862
S intrapariet and P trans (R)	0.7758	0.8862
G parietal sup (R)	0.7758	0.8862

Abbreviations: Inferior part of the precentral sulcus (S precentral-inf-part); superior part of the precentral sulcus (S precentral-sup-part); middle-anterior part of the cingulate gyrus and sulcus (G and S cingul-Mid-Ant); intraparietal sulcus and transverse parietal sulci (S intrapariet and P trans); superior parietal lobule (G parietal sup); left (L); right (R).

Table S4. Association of 2-back accuracy and cognition score with the ROIs activity for the contrasts of face versus place and emotional versus neutral face.

Regions	Two-back accuracy		Cognition score	
	Face versus place contrast	Emotional versus neutral face contrast	Face versus place contrast	Emotional versus neutral face contrast
	P_{FDR}	P_{FDR}	P_{FDR}	P_{FDR}
S precentral-inf-par (L)	0.1678	0.0787	0.9761	0.6238
S precentral-sup-part (L)	0.4356	0.0883	0.9761	0.5420
G and S cingul-Mid-Ant (L)	0.2488	0.1567	0.9761	0.6238
S intrapariet and P trans (L)	0.9671	0.1271	0.9941	0.4329
G parietal sup (L)	0.9273	0.2367	0.9761	0.4329
S precentral-inf-par (R)	0.1890	0.0787	0.9761	0.6238
S precentral-sup-part (R)	0.2687	0.2367	0.9761	0.6238
G and S cingul-Mid-Ant (R)	0.6381	0.2367	0.9761	0.5420
S intrapariet and P trans (R)	0.9671	0.0787	0.9761	0.4329
G parietal sup (R)	0.9671	0.2367	0.9761	0.4765

Table S5. The coefficients in the mediation model testing whether the ROIs activity for the 2-back versus 0- back contrast mediated the effect of sleep duration on 2-back accuracy.

	Path a		Path b		Path c'		Path c		Path a*b	
	β	P_{FDR}	β	P_{FDR}	β	P_{FDR}	β	P_{FDR}	β	P_{FDR}
S precentral-sup-part (L)	0.0394	0.0095	0.0927	0.0003	0.0653	0.0002	0.0689	0.0002	0.0037	0.0068
S intrapariet and P trans (L)	0.0414	0.0064	0.1786	0.0003	0.0615	0.0002	0.0689	0.0002	0.0074	0.0059
G parietal sup (L)	0.0522	0.0011	0.0706	0.0003	0.0652	0.0002	0.0689	0.0002	0.0037	0.0007
S precentral-sup-part (R)	0.0494	0.0011	0.0864	0.0003	0.0646	0.0002	0.0689	0.0002	0.0043	0.0007
S intrapariet and P trans (R)	0.0425	0.0064	0.1908	0.0003	0.0608	0.0002	0.0689	0.0002	0.0081	0.0059
G parietal sup (R)	0.0432	0.0064	0.0932	0.0003	0.0649	0.0002	0.0689	0.0002	0.0040	0.0059

Table S6. The coefficients in the mediation model testing whether the ROIs activity for the 2-back versus 0- back contrast mediated the effect of sleep duration on cognition score.

	Path a		Path b		Path c'		Path c		Path a*b	
	β	P_{FDR}	β	P_{FDR}	β	P_{FDR}	β	P_{FDR}	β	P_{FDR}
S precentral-sup-part (L)	0.0394	0.0096	0.0869	0.0002	0.0454	0.0069	0.0488	0.0027	0.0034	0.0074
S intrapariet and P trans (L)	0.0414	0.0062	0.1556	0.0002	0.0423	0.0072	0.0488	0.0027	0.0064	0.0058
G parietal sup (L)	0.0522	0.0009	0.0762	0.0002	0.0448	0.0069	0.0488	0.0027	0.0040	0.0006
S precentral-sup-part (R)	0.0494	0.0023	0.0669	0.0002	0.0455	0.0069	0.0488	0.0027	0.0033	0.0010
S intrapariet and P trans (R)	0.0425	0.0058	0.1386	0.0002	0.0429	0.0072	0.0488	0.0034	0.0059	0.0043
G parietal sup (R)	0.0432	0.0058	0.0760	0.0002	0.0455	0.0069	0.0488	0.0027	0.0033	0.0043
